# The IRE1α-XBP1s pathway promotes insulin-stimulated glucose uptake in adipocytes by increasing PPARγ activity

**DOI:** 10.1038/s12276-018-0131-0

**Published:** 2018-08-15

**Authors:** Yoon Mi Cho, Dong Hee Kim, Kyung Hye Lee, Seong-Whan Jeong, Oh-Joo Kwon

**Affiliations:** Department of Biochemistry, College of Medicine, The Catholic University of Korea, Seoul, Republic of Korea

## Abstract

The peroxisome proliferator-activated receptor-γ (PPARγ) improves whole-body insulin sensitivity by regulating the adipogenic and metabolic functions of mature adipocytes. We have previously demonstrated that an active splice variant of X-box binding protein 1 (XBP1s) enhances PPARγ expression during adipogenesis. In this study, we investigated the role of XBP1s, particularly with respect to PPARγ, in the mechanisms underlying insulin sensitivity in mature adipocytes. Insulin was able to stimulate XBP1s generation by activating inositol-requiring enzyme 1 (IRE1) α and was also able to increase its transcriptional activity by inducing nuclear translocation. XBP1s also upregulated the levels of phosphorylated IRS1 and AKT, demonstrating a positive feedback regulatory mechanism linking insulin and XBP1s. XBP1s enhanced the expression of fibroblast growth factor 21 and, in turn, increased PPARγ activity, translocation of GLUT4 to the cell surface, and glucose uptake rate in adipocytes. In addition, XBP1s abolished palmitate-induced insulin resistance in adipocytes by increasing adiponectin secretion, repressing the secretion of pro-inflammatory adipokines such as leptin, monocyte chemoattractant protein 1, and tumor necrosis factor α, and decreasing fatty acid release. These findings provide a novel mechanism by which XBP1s stimulate insulin sensitivity in adipocytes through fibroblast growth factor 21 induction and PPARγ activation.

## Diabetes: Restoring insulin sensitivity

Researchers have identified a protein, XBP1s, that may help treat type II diabetes by re-sensitizing cells to insulin. Insulin controls blood sugar levels by triggering cells to absorb sugar from the blood. In obese individuals, cells can lose sensitivity to insulin, requiring increasing quantities to trigger sugar uptake, disrupting blood sugar regulation. Termed insulin resistance, this is a major risk factor for type II diabetes and other diseases. XBP1s was previously known to affect insulin sensitivity, but the mechanism was unclear. Oh-Joo Kwon and co-workers at The Catholic University of Korea in Seoul investigated how XBP1s affected the response of mouse fat cells to insulin. They found that XBP1s restored insulin sensitivity, turning insulin-resistant cells into cells that responded to insulin by absorbing sugar. XBP1s may be useful in treatment or prevention of type II diabetes.

## Introduction

Adipose tissue is a key energy storage organ and is also becoming known as an endocrine organ with multiple roles in metabolism and the regulation of whole-body energy homeostasis^1^. In conditions characterized by excessive energy, such as obesity, insulin resistance is locally initiated in adipose tissue and subsequently progresses to systemic insulin resistance. This systemic resistance is thought to be a major risk factor for metabolic disorders such as type 2 diabetes, fatty liver disease, atherosclerosis, and some cancers^2^. Consequently, understanding how insulin sensitivity is regulated in adipocytes may be of therapeutic value for the treatment of obesity-related metabolic diseases.

Peroxisome proliferator-activated receptor-γ (PPARγ) is a ligand-activated transcription factor that belongs to the nuclear receptor superfamily. PPARγ is most abundantly expressed in adipose tissue, and both its expression and activity are regulated by insulin^3^, suggesting that PPARγ functions within adipose tissue to regulate insulin sensitivity. PPARγ activation exerts systemic insulin-sensitizing effects by inducing the expression of several genes involved in the insulin signaling cascade as well as the synthesis of beneficial adipokines within adipose tissue^4^. In addition, PPARγ is widely accepted as a master regulator of adipocyte differentiation (adipogenesis), an important mechanism for maintaining insulin sensitivity in adipose tissue and preventing ectopic fat accumulation and cardiovascular risk^5^. Therefore, synthetic PPARγ ligands are widely used in the treatment of insulin resistance-related pathological conditions^6^; however, the detailed mechanisms underlying PPARγ activation in adipose tissue are poorly understood.

X-box binding protein 1 (XBP1) is a member of the bZIP family of transcription factors. XBP1 is converted to its transcriptionally active isoform (XBP1s) by inositol-requiring enzyme 1 (IRE1) α-mediated unconventional splicing of XBP1 mRNA^7^. Most of the knowledge regarding the role of XBP1s in insulin sensitivity comes from in vitro studies of adipogenesis. In this context, we have also previously identified a set of pro-adipogenic mechanisms by which XBP1s stimulates PPARγ2 expression directly and indirectly by regulating Wnt10b and miR-148a, respectively^8–10^. Findings from a relatively thorough in vitro investigation suggest that XBP1s plays a pro-adipogenic role. However, its role in mature adipocytes, which make up almost 30% of the cellular content of adipose tissue, is widely unknown. In relation to insulin signaling, a number of in vivo studies suggest that XBP1s is implicated in the regulation of whole-body insulin sensitivity. For example, both the nuclear translocation and activity of XBP1s are reduced in the livers of obese mice^11^; this decrease in XBP1s activity is associated with the development of insulin resistance and type 2 diabetes in obese mice^12^, while enhancing XBP1s activity increases hepatic insulin sensitivity and improves glucose homeostasis^12,13^. However, the adipose-specific roles of XBP1s have not been fully addressed. To the best of our knowledge, only two studies have demonstrated adipose-specific in vivo roles of XBP1s, and these findings have not been consistent with each other. One study reported that adipocyte-specific deletion of XBP1s did not affect inflammation or metabolic homeostasis^14^. In contrast, a second study reported that XBP1s improves systemic glucose homeostasis by increasing adiponectin multimerization in an adipocyte-specific XBP1s gain-of-function mouse model^15^. Further studies are needed to elucidate the adipocyte-specific role of XBP1s in the regulation of insulin sensitivity.

In this study, we explored the insulin-sensitizing role of XBP1s and the underlying molecular mechanisms of this sensitization in differentiated mouse 3T3-L1 adipocytes. This study provides new insights into the role of XBP1s in the pathogenesis and treatment of metabolic disorders related to insulin resistance.

## Materials and methods

### Culture and differentiation of 3T3-L1 cells

Murine 3T3-L1 fibroblasts (American Type Culture Collection, Manassas, VA, USA) were maintained and differentiated into adipocytes as previously described^9^. Briefly, cells were maintained for 2 days in 4.5 g l ^−1^ glucose-containing Dulbecco's modified Eagle's medium (DMEM) supplemented with 10% normal calf serum (Gibco, Grand Island, NY, USA). Two days after reaching confluence (day 0), adipogenic differentiation was induced for 2 days in medium containing insulin (1 μg ml^−1^; Sigma, St Louis, MO, USA), IBMX (0.5 mM; Sigma), and dexamethasone (1 μM; Sigma). Then, the cells were cultured for 2 days in DMEM supplemented with 10% fetal bovine serum (FBS) (Gibco) and insulin (1 μg ml^−1^). Following this, the medium was changed to the DMEM/10% FBS until day 8.

### Genetic manipulation of XBP1s and FGF21 in adipocytes

Differentiated 3T3-L1 adipocytes were transiently transfected by electroporation as follows. Cells were detached from the plates with trypsin/EDTA and resuspended in serum-free Opti-MEM (Gibco) prior to electroporation. Approximately 2.5 million cells were mixed with 20 nM XBP1 (sense: GACCAUCACAUUGCCUAGA, antisense: UCUAGGCAAUGUGAUGGUC) or fibroblast growth factor 21 (FGF21; sense: AGAUGACGACCAAGACACU, antisense: AGUGUCUUGGUCGUCAUCU) siRNA duplexes (Bioneer, Daejeon, Korea) or 1 μg of a mouse XBP1s-overexpressing recombinant plasmid (pcDNA3.1-mXBP1s)^9^. Then, the cells were pulsed at 1300 V for 20 ms using the Neon™ Transfection System (Invitrogen, Carlsbad, CA, USA) and were immediately incubated with fresh media before reseeding. Experiments were conducted 48 h after electroporation.

### Measurement of glucose uptake rate

3T3-L1 adipocytes were cultured overnight in serum-free DMEM in 12-well plates and then washed in phosphate-buffered saline (PBS) and incubated in glucose-free DMEM for 1 h. Subsequently, 18.5 kBq of 2-deoxy-d-^3^H-glucose (PerkinElmer, San Jose, CA, USA) and 0.1 mM 2-deoxyglucose were added. After 30 min, the reaction was terminated with cytochalasin B (20 μM), and the cells were washed with ice-cold PBS. Cells were lysed for 10 min in 0.1% sodium dodecyl sulfate (SDS), and then aliquots of lysates were used for liquid scintillation counting and determination of protein concentration by BCA protein assay (Thermo Scientific, Rockford, IL, USA). Radioactivity was measured using a β-counter (PerkinElmer). Radioactivity was normalized to protein concentration and is presented as a percentage of basal level.

### Immunofluorescence microscopic examination

3T3-L1 adipocytes were grown and differentiated on coverslips and then fixed with 4% paraformaldehyde for 20 min. After washing with PBS for 10 min, cells were blocked with 2% bovine serum albumin (BSA) in PBS for 30 min. After washing again, cells were incubated with rabbit anti-mouse GLUT4 antibody (Merck Millipore, Darmstadt, Germany, #07-1404) (1:100 dilution in 1% BSA/PBS) overnight at 4 °C. Cells were then washed and incubated for 1 h at room temperature with FITC-conjugated anti-rabbit IgG (1:150 in 1% BSA/PBS). Nuclei were stained with DAPI (Invitrogen), and the images were visualized with an LSM 510 Meta confocal laser-scanning microscope (Zeiss, Oberkochen, Germany) at ×400 magnification.

### Real-time PCR

Total RNA was extracted using TRIzol reagent (Invitrogen), and complementary DNA was synthesized using the PrimeScript RT kit (TaKaRa, Shiga, Japan). Real-time PCR analysis was performed using an ABI 7300 thermal cycler (Applied Biosystems, Foster City, CA, USA) with SYBR Green Q-PCR Master Mix (TaKaRa). The ΔΔCt method was used to calculate the relative mRNA levels, using the 36b4 gene as an internal control. The primer sequences used for PCR are listed in Table 1.

**Table 1:** Primer sequences for PCR analysis

Name	Primer sequences (5'–3')		Size (bp)	Gene ID
	Sense	Antisense		
Adiponectin	TGTGTATCGCTCAGCGTTCAGTGT	AGAGAACGGCCTTGTCCTTCTTGA	224	NM_009605
aP2	GAT GCC TTT GTG GGA ACC T	CTG TCG TCT GCG GTG ATT T	230	NM_024406
CD36	CAG GTC TAT CTA CGC TGT GTT C	CAG GCT TTC CTT CTT TGC ATT T	200	NM_007643
ERdj4	AGT CTG CCT CAG AGC GAC AAA TCA	GCC TCT TTG TCC TTT GCC ATT GGT	203	NM_013760
FGF21	CTACACAGATGACGACCAAGA	ATCAAAGTGAGGCGATCCATAG	205	NM_020013
GLUT4	TCG TGG CCA TAT TTG GCT TTG TGG	TAA GGA CCC ATA GCA TCC GCA ACA	190	NM_009204
Leptin	GGC TTT GGT CCT ATC TGT CTT AT	ATT CTC CAG GTC ATT GGC TAT C	278	NM_008493
LPL	ACGAGCGCTC CATTCATCTC TTCA	TCTTGCTGCT TCTCTTGGCT CTGA	180	NM_008509
MCP1	GAA GGA ATG GGT CCA GAC ATA C	TCA CAC TGG TCA CTC CTA CA	200	NM_011333
p58^IPK^	TTT CAC GCT GCA GTT GAT GGT GAC	TCT TCT GCT TCG TCA AGC TTC CCT	200	NM_008929
PPARγt	CGA GCC CTG GCA AAG CAT TTG TAT	AAA TTC GGA TGG CCA CCT CTT TGC	211	NM_001308354
PPARγ2	CGCTGATGCACTGCCTATGA	AGAGGTCCACAGAGCTGATTCC	101	NM_011146
TNF	CGTCAGCCGATTTGCTATCT	AGCAATGACTCCAAAGTAGACC	232	NM_013693
XBP1t	AGC AGC AAG TGG TGG ATT TGG AAG	AAG AGG CAA CAG TGT CAG AGT CCA	299	NM_013842
XBP1s	CTGAGGTCCGCAGCAGGT	TGTCAGAGTCCATGGGAAGA	66	NM_001271730
36B4	GCTCCAAGCAGATGCAGCA	CCGGATGTGAGGCAGCAG	143	NM_007475
fgf21 promoter (ChIP assay)	CTC AGA CCA AGG AGC ACA GA	TGA ACG CAG AAA TAC CAG AAT	257	ENSMUST00000033099
pEGFP-mXBP1	TTG AAT TCT GAT GGT GGT GGT GGC AGC GGC GCC GA	TTG TCG ACA GGC CTG CAC CTG CTG CAG AGG TGC ACA	–	–
pcDNA-WT-mXBP1u	TTG CGG CCG CAT GGT GGT GGT GGC A	TTA AGC TTG ACA CTA ATC AGC TGG GGG AAA AGT TC	**–**	**–**
pcDNA-Mut-mXBP1u	ACT CAG ACT ATG TGC ACC TTT GCG ACA GGT GCA GGC CCA GTT	AAC TGG GCC TGC ACC TGT CGC AAA GGT GCA CAT AGT CTG AGT	**–**	**–**

### In vitro insulin resistance model

To establish an insulin-resistant state in vitro, cells were pretreated with low serum-containing media (0.25% FBS) overnight, washed with PBS, and then incubated with palmitate (200 μM, Sigma) for 24 h. Palmitate-stimulated lipolysis was determined by measuring free glycerol content in the culture media using a serum triglyceride determination kit (Sigma) as described previously^9^. Briefly, the culture media was centrifuged at 16,000 *g* for 10 min, and 10 μl of glycerol standard or sample was mixed with 500 μl of kit reagents (Sigma). Glycerol concentration was measured at 540 nm and normalized to total cellular protein.

### Enzyme-linked immunosorbent assay

Media was collected and centrifuged for 10 min at 800 *g*. The supernatant fraction was stored at −70 °C. Adiponectin, leptin, tumor necrosis factor-α (TNFα), and MCP1 concentrations were determined using a Multiplex MAP Mouse Adipocyte Panel (Merck Millipore).

### Preparation of cytoplasmic and nuclear fractions

Cells were washed twice with ice-cold PBS, incubated at 4 °C for 5 min in hypotonic buffer (10 mM Tris-HCl, pH 7.6, 10 mM NaCl, 3 mM MgCl_2_, 0.5% NP-40, and protease inhibitors), scraped from the dish, and centrifuged at 130 *g* for 5 min. The supernatant was used as the cytoplasmic fraction. The pellet was resuspended in nuclear extraction buffer (0.4 M NaCl, 5 mM HEPES, pH 7.9, 26% glycerol, 1.5 mM MgCl_2_, 0.2 mM EDTA, 0.5 mM dithiothreitol, and protease inhibitors), incubated at 4 °C for 30 min, and then centrifuged at 30,000 *g* for 20 min. The supernatant resulting from this step was used as the nuclear fraction.

### Immunoblot analysis

Cells were lysed in RIPA buffer and 30 μg of each protein sample was separated using 10% SDS-polyacrylamide gel electrophoresis and transferred onto a nitrocellulose membrane (Schleicher & Schuell, Dassel, Germany). The membranes were incubated with mouse anti-GAPDH (1:500; Santa Cruz Biotechnology, Dallas, TX, USA), goat anti-Lamin B (1:500; Santa Cruz), or rabbit antisera against XBP1 (1:200; BioLegend, San Diego, CA, USA), GLUT4 (1:500; Chemicon, Temecula, CA, USA), PPARγ (1:500; Santa Cruz), phospho-insulin receptor (P-IR) (1:500; Cell Signaling, Danvers, MA, USA), IR (1:500; Cell Signaling), phospho-insulin receptor substrate 1 (P-IRS1) (1:500; Cell Signaling), IRS1 (1:500; Cell Signaling), phospho-AKT (P-AKT; 1:500; Epitomics, Burlingame, CA, USA), AKT (1:500; Epitomics), NFκB (1:500; Cell Signaling), phospho-JNK (P-JNK; 1:500; Cell Signaling), JNK (1:500; Cell Signaling), FGF21 (1:500; Cusabio, College Park, MD, USA), phospho-IRE1α (P-IRE1α; 1:500; Novus Biologicals, Littleton, CO, USA), and IRE1α (1:500; Novus Biologicals). Antibody binding was detected using horseradish peroxidase-conjugated anti-rabbit, anti-mouse (1:2,000; Sigma), or anti-goat IgG (1:2000; Santa Cruz), and the immunoreactive bands were visualized using the ECL method (Amersham Pharmacia, Buckinghamshire, UK). For statistical analysis, immunoreactivity was quantified by densitometry using a Gel Doc EZ System (Bio-Rad, Hercules, CA, USA).

### Chromatin immunoprecipitation assay

Cells were cross-linked with formaldehyde and then sonicated to generate 200–1000 bp DNA fragments. The fragmented chromatin samples were immunoprecipitated with rabbit anti-mouse XBP1 (5 μg; Santa Cruz), reverse cross-linked, purified, and analyzed by PCR according to the supplier’s protocol (Merck Millipore). Primers for mouse *fgf21* promoter region amplicons are listed in Table 1. DNA samples that were immunoprecipitated with rabbit IgG were used as negative controls.

### Assays for XBP1 mRNA splicing

To detect the activation of the IRE1/XBP1s pathway in mature adipocytes, we generated an XBP1-EGFP fusion reporter construct (pEGFP-mXBP1). The XBP1 coding sequence was amplified using reverse transcriptase (RT)-PCR, digested with *EcoR*I and *Sal*I enzymes, and then subcloned into a pEGFP-N1 vector (Invitrogen). Subsequently, the EGFP coding region was inserted into the putative IRE1α splice site, giving rise to a fusion protein that lacks the XBP1 C-terminal region. EGFP is out of frame without IRE1α-mediated splicing, but is in frame with XBP1 after the 23-base stem–loop of XBP1 is eliminated by splicing. Cells were transfected with pEGFP-mXBP1 and fixed for fluorescence microscopy. The green fluorescent area and intensity was quantified with ImageJ.

Next, to measure functional XBP1s activity, we generated an expression construct in which full-length wild-type unspliced XBP1 (XBP1u) gene is inserted (pcDNA-WT-mXBP1u). XBP1u was amplified by RT-PCR, digested with *Not*I and *Bam*HI enzymes, and subcloned into pcDNA3.1 vector (Invitrogen). To generate the mutation construct (pcDNA-Mut-mXBP1u), the 4-base putative IRE1α splicing site was mutagenized using a QuikChange Site-Directed Mutagenesis Kit (Stratagene, La Jolla, CA, USA) (EMM 2016 48:e226). These two constructs were transfected into cells by electroporation. To examined the IRE1α spedicificty of XBP1 mRNA splicing, cells were pretreated with the IRE1α inhibitor STF-083010 (50 μM, Sigma) for 1 h.

### PPARγ activity assay

The PPARγ responsive element reporter was generated by inserting three copies of the DR1 sequence (TTCTGACCTATGACCTGG) into a pGL3-basic reporter plasmid (pGL3-DR1). Cells (2 × 10^4^ per well in 24-well plates) were co-transfected with pGL3-DR1 (50 ng) and either siRNA (20 nM) or plasmid (500 ng). Cells were then treated with reagents including PPARγ activator (rosiglitazone, 1 μM) (Sigma) or inhibitor (GW9662, 1 μM) (Sigma), IRE1α inhibitor STF-083010 (50 μM), or palmitate (200 μM) as indicated. After 48 h, cells were lysed and luciferase activity was measured using a Dual Luciferase Assay Kit (Promega, Madison, WI, USA). Transfection efficiency was normalized using the renilla-expressing plasmid (pRL-CMV-luc; 5 ng).

### Statistical analysis

All values are expressed as the means ± SEM from at least three independent experiments. The data were analyzed using Student’s *t*-test or one- or two-way analysis of variance followed by Tukey’s multiple comparison test. Statistical analyses were performed using GraphPad Prism 5 for Windows (GraphPad Prism Software, La Jolla, CA, USA). In all cases, statistical significance was accepted at *P* < 0.05.

## Results

### XBP1s enhances insulin-stimulated glucose uptake in adipocytes

**Fig. 1:**
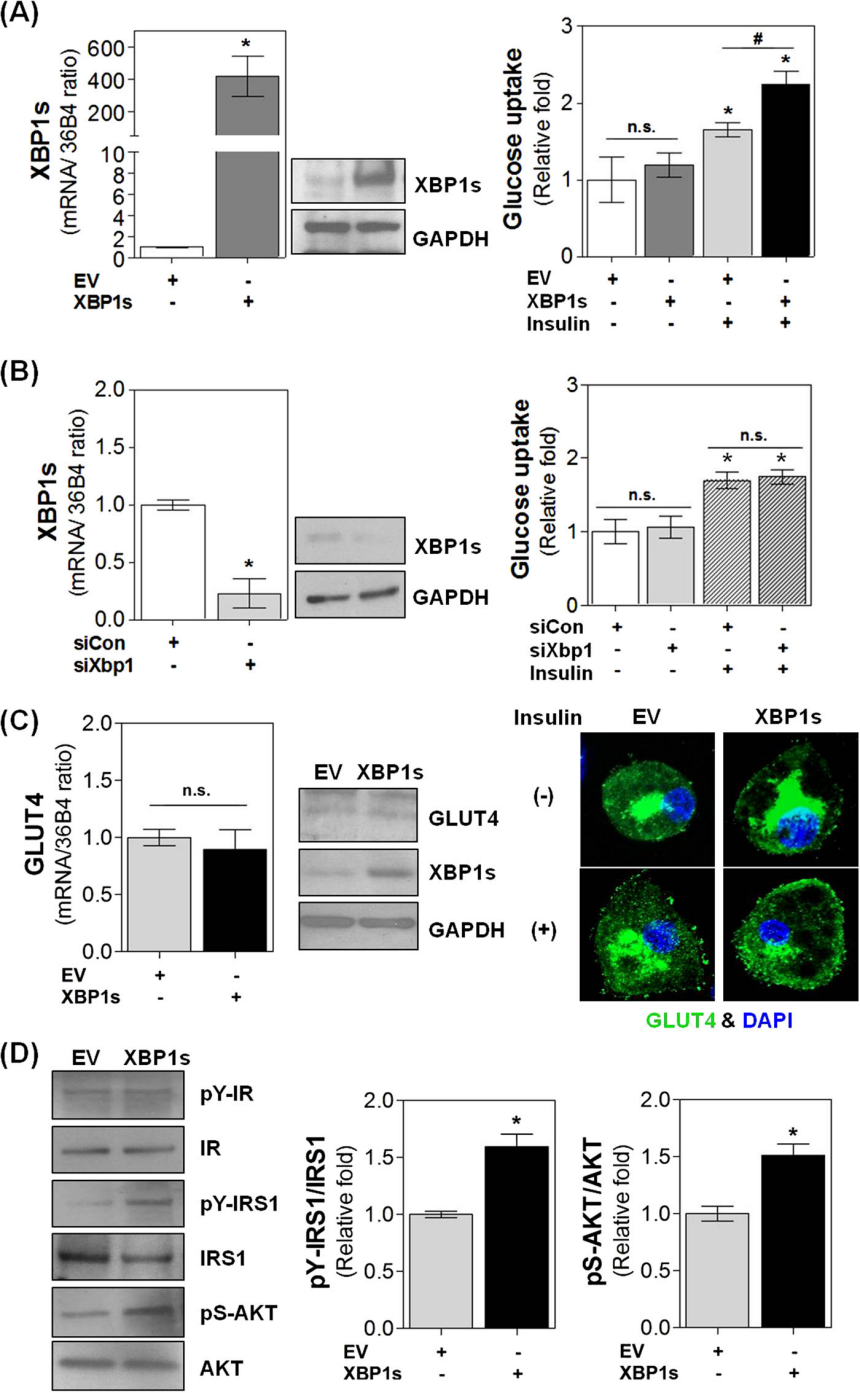
Effect of XBP1s on insulin-stimulated glucose uptake in 3T3-L1 adipocytes. 3T3-L1 adipocytes were transfected with pcDNA3.1-mXBP1s (XBP1s) or an siRNA against XBP1 (siXBP1), and treated with insulin (1 μg ml^−1^) for 30 min as indicated. **a**, **b** Intracellular XBP1s mRNA and protein levels and glucose uptake rates were determined. **c** The expression (left) and translocation (right, green) of GLUT4 were analyzed using real-time PCR/immunoblot analyses and immunofluorescence, respectively. DAPI was used to stain nuclei (right, blue). **d** The total and phosphorylated expression levels of insulin receptor (IR), insulin receptor substrate 1 (IRS1), and AKT were examined by immunoblot analysis (left) and quantified with densitometry (right). Values are presented as the means ± SEM of three independent experiments. ^*^P < 0.05 compared to empty vector (EV) or control siRNA (siCon); ^#^*P* < 0.05 compared to insulin-treated EV. n.s. not significant

The major physiological action of insulin on glucose homeostasis is to stimulate the cellular uptake of glucose in insulin-sensitive organs, such as skeletal muscle and adipose tissue. To examine the role of XBP1s in insulin sensitivity in adipocytes, we first measured the rate of insulin-dependent glucose uptake in XBP1s-overexpressing mouse adipocytes (Fig. 1a). In the absence of insulin, XBP1s did not increase the glucose uptake rate; however, in adipocytes treated with insulin (1 μg ml^−1^, 30 min) XBP1s significantly increased the glucose uptake rate. Additionally, glucose uptake was not reduced upon XBP1s knockdown regardless of the presence of insulin (Fig. 1b), indicating that XBP1s is expressed at very low levels in mature adipocytes^16^.

Next, we examined the effect of XBP1s on the expression of GLUT4, the principal insulin-sensitive glucose transporter in adipocytes^17,18^. Although XBP1s is a functionally active transcription factor, XBP1s overexpression did not increase GLUT4 expression at either the mRNA or protein levels, indicating that GLUT4 is not an XBP1s target gene (Fig. 1c, left). However, in the presence of insulin, XBP1s overexpression clearly increased GLUT4 translocation from the cytoplasm to the cell surface (Fig. 1c, right). It is well known that GLUT4 redistribution is regulated by insulin receptor signaling pathways;^17,18^ therefore, we examined whether XBP1s is involved in the activation of insulin signaling in adipocytes. As shown in Fig. 1d, XBP1s significantly elevated insulin receptor-mediated phosphorylation of IRS1 and AKT (Fig. 1d) in insulin-treated adipocytes, suggesting that XBP1s amplifies insulin signaling in adipocytes. Taken together, these findings implicate XBP1s as a putative positive regulator of insulin sensitivity in mature adipocytes.

### XBP1s increases insulin-mediated PPARγ activity in adipocytes

**Fig. 2:**
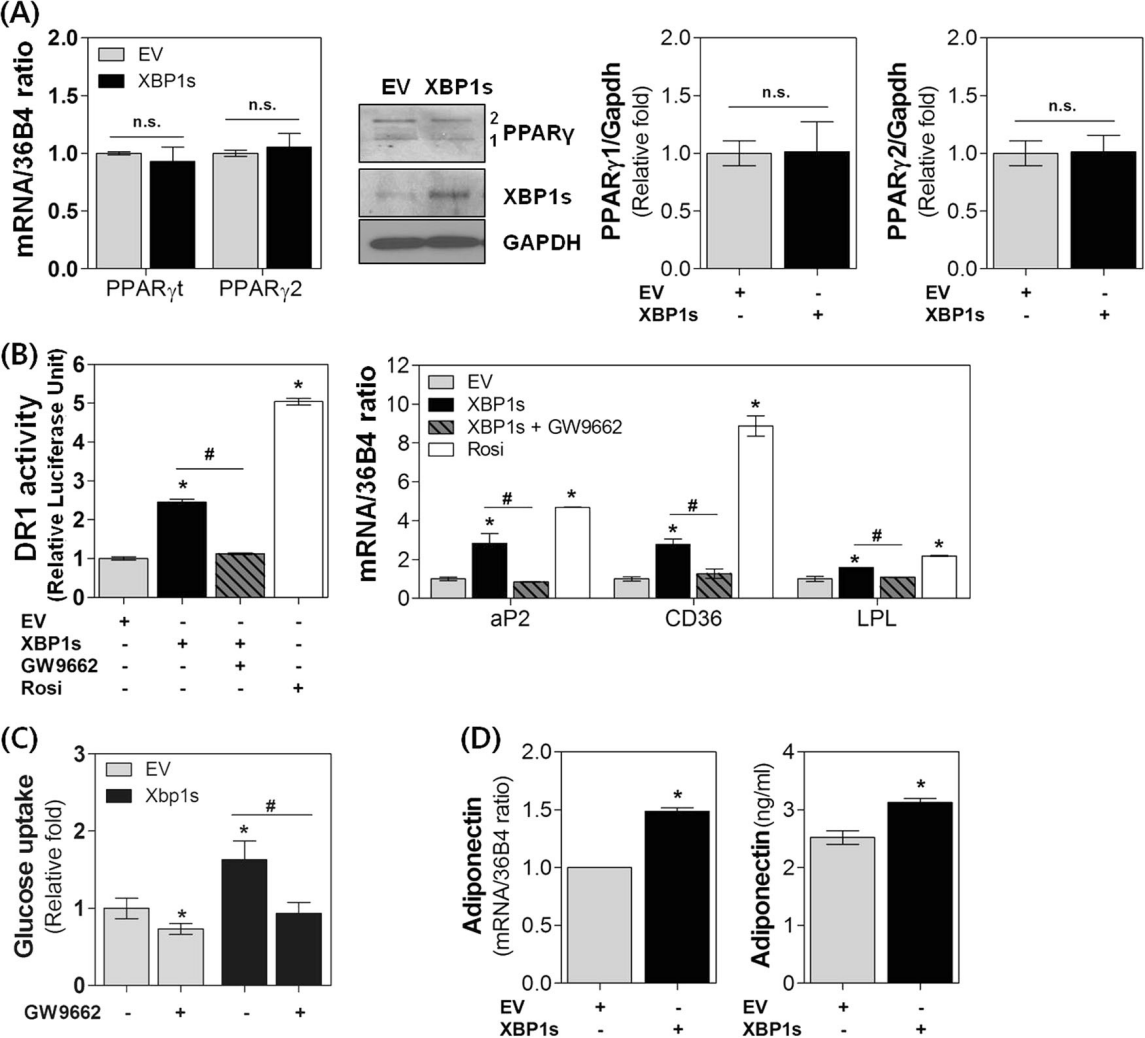
XBP1s enhances insulin-mediated PPARγ activation in adipocytes. 3T3-L1 adipocytes were transfected with control vector (EV) or pcDNA3.1-mXBP1s (XBP1s) and treated for 30 min with insulin (1 μg ml^−1^). **a** XBP1s and PPARγ (1 and 2, or total) mRNA and protein levels were analyzed by real-time PCR and/or immunoblotting (left) and quantified with densitometry (right). **b** The effect of an XBP1s and PPARγ activator (rosiglitazone, Rosi) or inhibitor (GW9662) on DR1 promoter activity (left) and the expression of the PPAR**γ** target genes aP2, CD36, and LPL (right) were examined by real-time PCR. **c** The effect of GW9662 on the XBP1s-mediated increase in glucose uptake was measured. **d** The effect of XBP1s on adiponectin mRNA and secreted protein levels were determined using real-time PCR and ELISA, respectively. Values are presented as the means ± SEM of three independent experiments. ^*^P < 0.05 compared to EV. ^#^*P* < 0.05 compared to XBP1s. n.s. not significant

Adipose tissue is the major mediator through which PPARγ affects insulin sensitivity. Activation of adipose PPARγ induces the expression of several genes encoding proteins involved in the insulin signaling cascade, thereby increasing insulin-mediated glucose uptake and regulating the release of adipokines^19^. In our previous reports, we indicated that XBP1s is a principal regulator of PPARγ transcription during adipogenesis^8–10^. Accordingly, we first examined the effect of XBP1s on PPARγ expression in mature adipocytes. However, in contrast to its effect in preadipocytes, XBP1s overexpression did not increase PPARγ expression at either the mRNA (total and PPARγ2 isoforms) or protein (PPARγ1 and PPARγ2) levels (Fig. 2a). In contrast, XBP1s overexpression significantly increased PPARγ activity by approximately 2.5-fold. Treatment of adipocytes with the PPARγ antagonist GW9662 almost completely suppressed XBP1s-induced PPARγ activity and mRNA levels of typical PPARγ target genes such as Ap2 and CD36 (Fig. 2b). The effect of XBP1s on insulin-stimulated glucose uptake was also significantly decreased upon GW9662 treatment (Fig. 2c). In addition, XBP1s increased the expression and secretion of adiponectin (Fig. 2d), an adipokine that is a known target of PPARγ^20^ and a well-known insulin-promoting protein that facilitates glucose uptake through increased GLUT4 translocation^21^. These results strongly suggest that the functional activation of endogenous PPARγ, and the resulting increase in adiponectin production, may be the principal mechanism by which XBP1s mediates increased cell surface translocation of GLUT4 and glucose uptake in mature adipocytes.

### FGF21 is a key regulator of XBP1s-mediated PPARγ activation in adipocytes

**Fig. 3:**
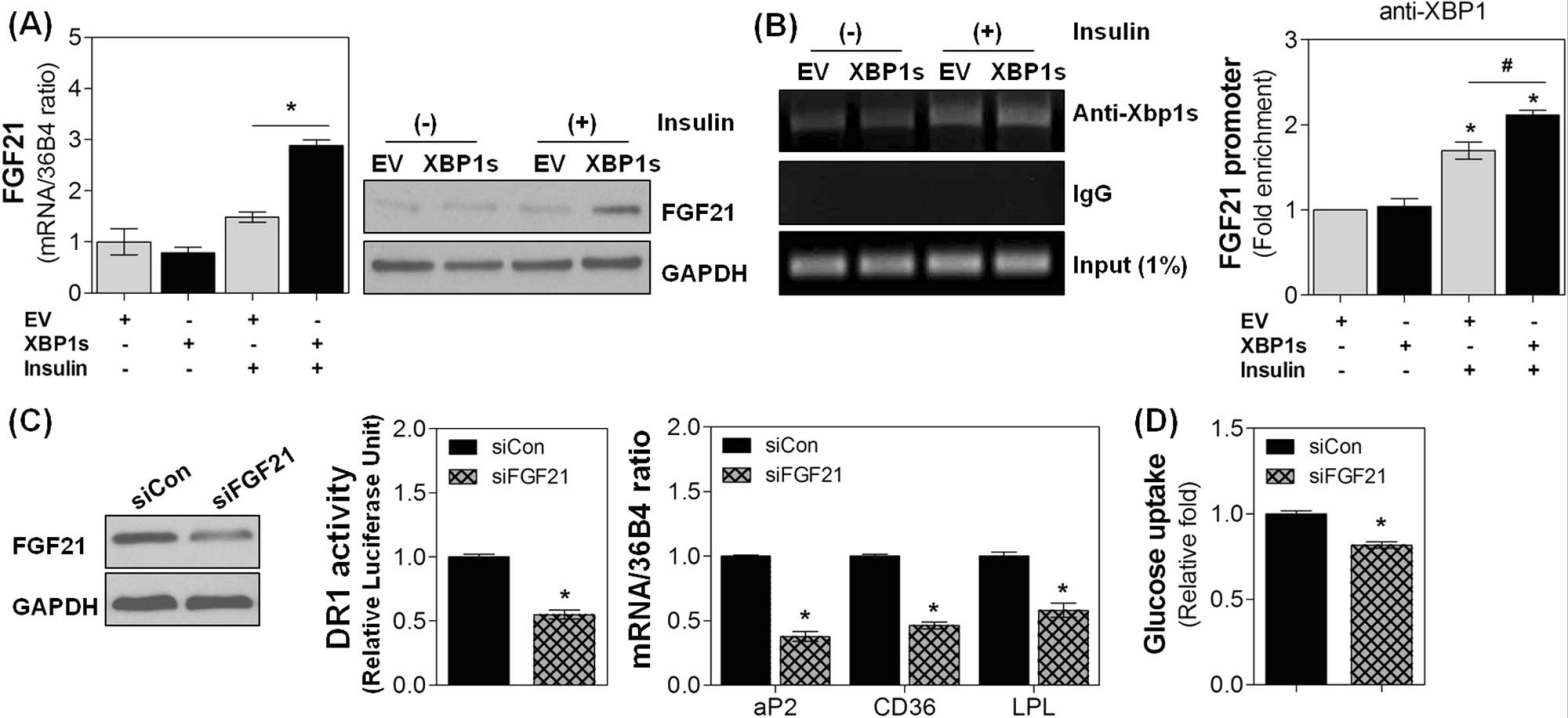
XBP1s promotes PPARγ activity through the induction of FGF21 in adipocytes. 3T3-L1 adipocytes were transfected with control vector (EV) or pcDNA3.1-mXBP1s (XBP1s) and treated for 30 min with insulin (1 μg ml^−1^) or vehicle as indicated. **a** FGF21 mRNA and protein levels were analyzed using real-time PCR and immunoblotting. **b** Chromatin immunoprecipitation was performed to determine the specific binding of XBP1s to the promoter region of *fgf21* using XBP1s antibodies (anti-XBP1s). The results were quantified by densitometry. **c**, **d** Effect of FGF21 knockdown on the XBP1-mediated activation of PPARγ activity and glucose uptake. XBP1s-overexpressing adipocytes were transfected with control (siCon) or siFGF21 (20 nM) and treated with insulin (1 μg ml^−1^, 30 min). DR1 promoter activity (**c**, middle), PPARγ target gene expression (**c**, right), and glucose uptake rate (**d**) were measured. Values are presented as the means ± SEM of three independent experiments. ^*^P < 0.05 compared to EV or siCon

As XBP1s is an active transcription factor, it can be easily inferred that other mediators that are transcriptionally induced by XBP1s may be involved in insulin-stimulated PPARγ activation in adipocytes. Among the known PPARγ activating proteins, FGF21 has been reported to be induced by XBP1s in hepatocytes^22^ and is suggested to be a potent activator of glucose uptake in adipose tissue^23–25^. We thus examined FGF21 as a putative target of XBP1s in the regulation of insulin sensitivity in mature adipocytes. XBP1s significantly increased the mRNA and protein levels of FGF21 only in the presence of insulin (Fig. 3a), and increased XBP1s enrichment was observed in the *fgf21* promoter region of insulin-treated adipocytes (Fig. 3b). Moreover, XBP1s-induced regulation of PPARγ, target gene expression (Fig. 3c), and glucose uptake (Fig. 3d) were significantly suppressed by FGF21 knockdown, suggesting that XBP1s-mediated transcriptional activation of FGF21 may play an important role in insulin-stimulated PPARγ activation and glucose uptake in adipocytes.

### Insulin upregulates the generation and nuclear translocation of XBP1s in adipocytes

**Fig. 4:**
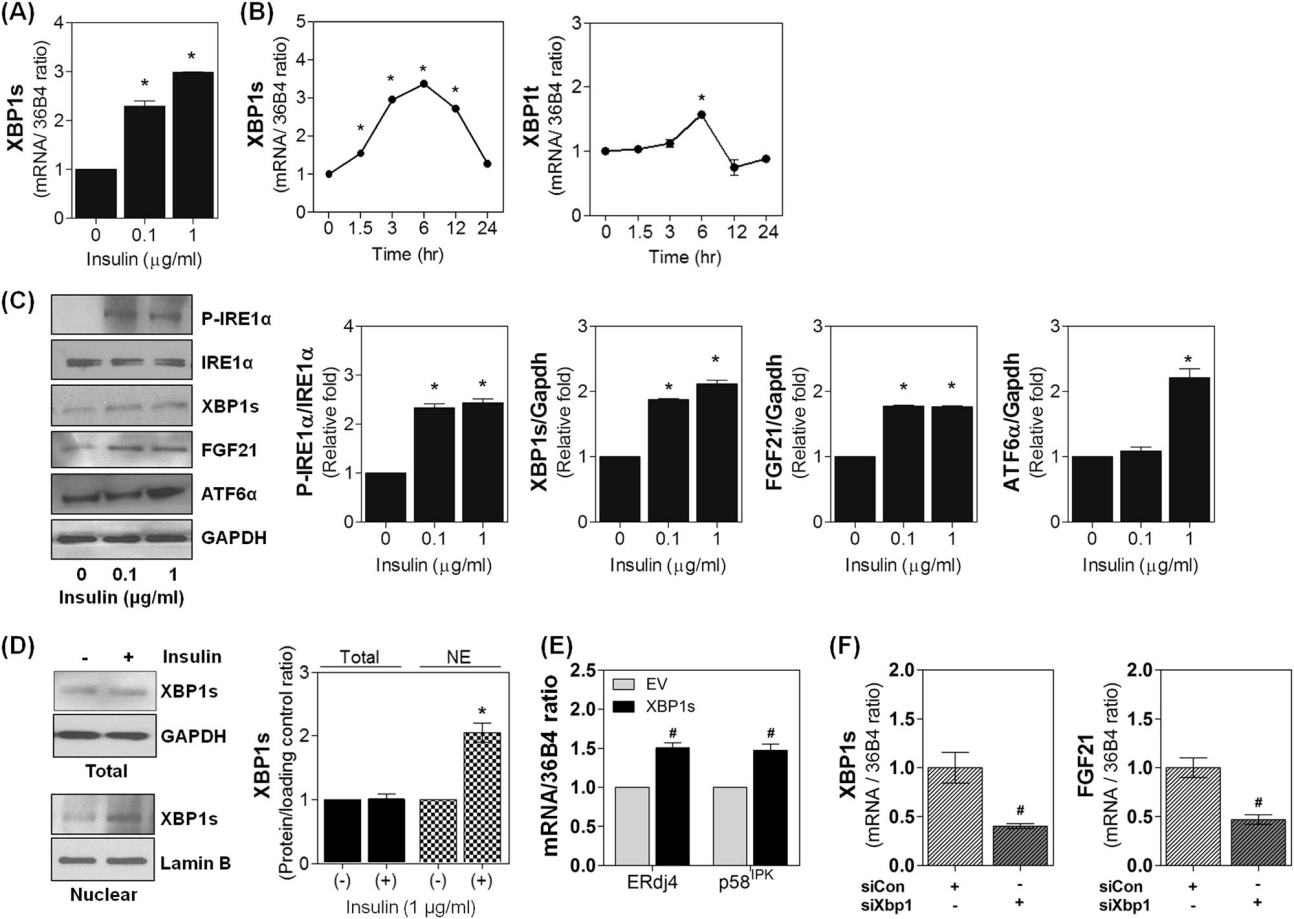
Insulin increases the generation and nuclear translocation of XBP1s in adipocytes. **a** 3T3-L1 adipocytes were treated for 6 h with the indicated dose of insulin, and mRNA levels of XBP1s were determined by real-time PCR. **b** The mRNA levels of spliced (XBP1s) or total XBP1 (unspliced+spliced, XBP1t) were determined by real-time PCR in 3T3-L1 adipocytes treated with insulin (1 μg ml^−1^) for the indicated time. **c** Dose-dependent effect of insulin on the levels of phosphorylated IRE1α (P-IRE1α), FGF21, XBP1s, and ATF6α were analyzed by immunoblotting and quantified with densitometry. **d**, **e** Insulin-mediated nuclear translocation and activation of XBP1s. XBP1s-overexpressing adipocytes were treated with vehicle or insulin (1 μg ml^−1^) for 30 min. **d** XBP1s expression levels in total cell lysates Total and nuclear fractions (NE) were examined by immunoblotting and densitometry analyses. GAPDH and Lamin B were used for loading controls. **e** Insulin-enhanced transcriptional activity of XBP1s was examined by measuring the mRNA expression levels of two XBP1s target genes, ERdj4 and p58^IPK^, using real-time PCR. **f** Role of XBP1s in insulin-mediated induction of FGF21. Adipocytes were transfected with control (siCon) or siXBP1 and treated with insulin (1 μg ml^−1^, 30 min). Then, XBP1s and FGF21 mRNA levels were determined. Values are presented as the means ± SEM of three independent experiments. ^*^P < 0.05 compared to insulin-untreated control. ^#^*P* < 0.05 compared to EV or siCon

Because our results (Fig. 1) and previous report^16^ showed that the basal level of XBP1s was too low to activate PPARγ activity, we postulated that insulin signaling might increase XBP1s levels in mature adipocytes. As shown in Fig. 4a, treatment with insulin (6 h) increased XBP1s mRNA levels in a dose-dependent manner. In time-course experiments, insulin (1 μg ml^−1^) induced XBPls transcription both rapidly and transiently; XBP1s mRNA levels increased beginning at 1.5 h, peaked at 6 h (3.3-fold increase), and returned to control levels by 24 h (Fig. 4b). XBP1s is produced by unconventional splicing of the XBP1 mRNA. To verify the involvement of insulin in XBP1 mRNA splicing, we examined the effect of insulin on the phosphorylation of IRE1α, an exclusive inducer of XBP1 posttranscriptional splicing. As shown in Fig. 4c, insulin strongly induced IRE1α phosphorylation as well as the expression of both XBP1s and FGF21 in a dose-dependent manner, suggesting that the specific activation of IRE1α is important for insulin-induced XBP1s production. Additionally, insulin increased total (unspliced plus spliced) levels of XBP1 mRNA 6 h after insulin treatment (Fig. 4b right), though this effect was much less potent than the effect of insulin on XBP1s levels. Correspondingly, the expression of ATF6α, a transcription factor responsible for the production of XBP1s, was also increased by insulin with the same temporal pattern (Fig. 4c). Taken together, these findings suggest that insulin may increase XBP1s mRNA levels principally by stimulating IRE1α-mediated splicing but not by stimulation XBP1 transcription.

With regard to the effect of insulin on XBP1s, recent reports have suggested that insulin promotes the nuclear translocation of XBP1s, thus increasing its transcriptional activity in kidney podocytes and the liver^12,26^. In our experiments using XBP1s-overexpressing adipocytes, insulin (1 μg ml^−1^, 30 min) significantly increased XBP1s nuclear translocation (Fig. 4d) as well as the expression of two target genes, ERdj4 and p58^IPK^ (Fig. 4e). In addition, the insulin-mediated increase in FGF21 expression in wild-type adipocytes was significantly reduced by XBP1s knockdown (Fig. 4f). Taken together, these findings indicate the functional importance of insulin-induced generation and activation of XBP1s in adipocytes.

### Physiological importance of insulin-mediated XBP1 mRNA splicing

**Fig. 5:**
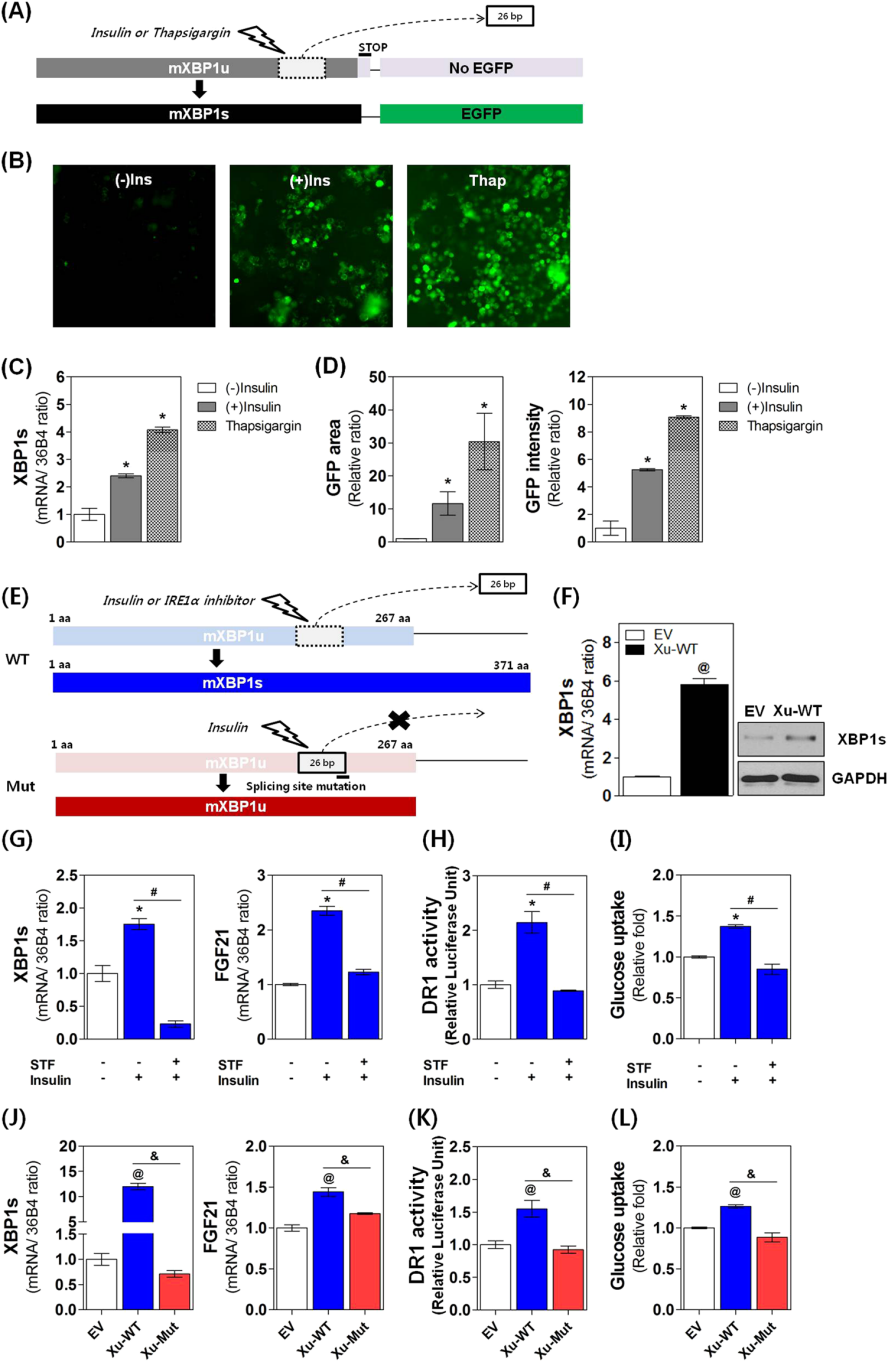
IRE1α-mediated XBP1 mRNA splicing is critical for insulin-stimulated glucose uptake in adipocytes. **a** Diagram of the pEGFP-mXBP1 reporter construct (see Materials and methods). **b**–**d** 3T3-L1 adipocytes were transfected with pEGFP-mXBP1 and treated for 6 h with insulin (1 μg ml^−1^) or thapsigargin (200 nM). The levels of GFP protein (**b**) and XBP1s mRNA (**c**) were examined using fluorescence microscopy and real-time PCR, respectively. GFP expression area and intensity were quantified with ImageJ (**d**). **e** Diagram of wild-type (pcDNA-WT-mXBP1u, upper) or mutant (pcDNA-Mut-mXBP1u, lower) constructs used for functional analyses of IRE1α-induced XBP1 splicing (see Materials and methods). **f**–**i** Adipocytes were transfected with pcDNA-WT-mXBP1u construct, pretreated for 1 h with vehicle or IRE1α inhibitor STF-083010 (STF, 50 uM), then treated with insulin (1 μg ml^−1^, 6 h) as indicated. **f** Effect of insulin on XBP1s mRNA and protein levels in adipocytes transfected with pcDNA-WT-mXBP1u. **g**–**i** Effect of STF-08310 on XBP1s and FGF21 mRNA levels, DR1 activity, and glucose uptake rate. **j**–**l** Effect of mutant construct (pcDNA-Mut-mXBP1u) on XBP1s and FGF21 mRNA levels, DR1 activity and glucose uptake rate. Values are presented as the means ± SEM of three independent experiments. ^*^P < 0.05 compared to the insulin-untreated group. ^#^*P* < 0.05 compared to the insulin-treated group. ^@^*P* < 0.05 compared to the EV-transfected group. ^&^*P* < 0.05 compared to the pcDNA-WT-mXBP1u-transfected group

IRE1α catalyzes unconventional mRNA splicing by removing an internal 26 nucleotides from the XBP1 mRNA. To confirm the involvement of IRE1α in the insulin-mediated increase in XBP1s production, we generated an XBP1-EGFP fusion construct designed to express EGFP only after the 26-base stem–loop of the XBP1 mRNA was spliced out by IRE1α endonuclease activity (Fig. 5a; also see Materials and methods). We then analyzed expression of the EGFP epitope upon insulin treatment. As shown in Fig. 5b, insulin treatment (1 μg ml^−1^, 24 h) significantly increased the area and the intensity of both EGFP expression (Fig. 5b, d) and XBP1s mRNA production (Fig. 5c). These findings indicate that IRE1α activation is a critical mechanism underlying insulin-induced XBP1s production.

To further examine the functional importance of insulin-stimulated splicing of mRNA through IRE1α activation, we developed the pcDNA-WT-mXBP1u construct in which the wild-type XBP1u gene was inserted. Thus, functional XBP1s protein could be produced only upon IRE1α activation (Fig. 5e upper; WT). In adipocytes transfected with pcDNA-WT-mXBP1u, both mRNA and protein XBP1s levels were significantly increased by insulin treatment (Fig. 5f), indicating the establishment of an insulin-mediated splicing system in adipocytes. In these cells, insulin (1 μg ml^−1^, 6 h) significantly increased FGF21 mRNA levels (Fig. 5g), DR1 activity (Fig. 5h), and glucose uptake (Fig. 5i). These insulin-mediated effects were completely reversed upon pretreatment with the IRE1α inhibitor STF-083010 (50 uM, 1 h). In addition, insulin-mediated effects almost disappeared in adipocytes transfected with a mutated construct, pcDNA-Mut-mXBP1u, in which functional XBP1s protein cannot be generated due to a 12 nt mutation in the IRE1α-recognizing site within the XBP1u mRNA (Fig. 5e, lower; Mut) (Fig. 5j–l). These findings implicate that insulin-mediated IRE1α activation in adipocytes is a critical step in the production of functional XBP1s as well as the subsequent increases in FGF21 levels, PPARγ activity, and glucose uptake.

### XBP1s attenuates palmitate-induced insulin resistance in adipocytes

**Fig. 6:**
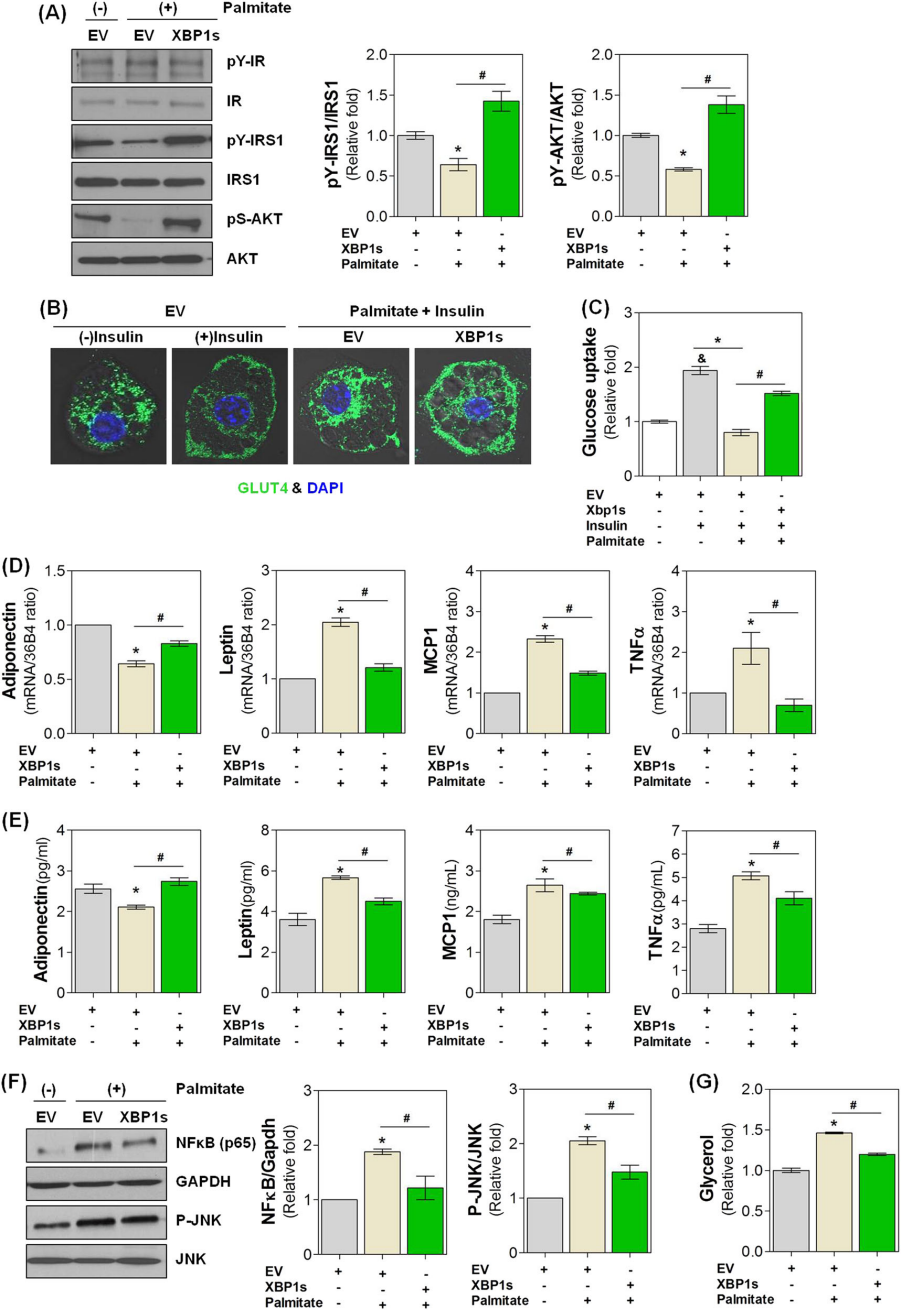
XBP1s attenuates palmitate-induced insulin resistance in adipocytes. Adipocytes were transfected with control (EV) or pcDNA3.1-mXBP1s (XBP1s) and treated with vehicle or palmitate (200 μM) for 24 h. Cells were further incubated with insulin (1 μg ml^−1^, 30 min) as indicated, and then the following parameters of insulin resistance were determined (see Materials and methods): **a** Insulin signaling; **b** GLUT4 distribution; **c** glucose uptake rate; **d**, **e** Intracellular mRNA and secreted protein levels of adipokines such as adiponectin, leptin, MCP1, and TNFα; **f** NFκB and P-JNK expression; and **g** glycerol release rate. Values are presented as the means ± SEM of three independent experiments. ^*^P < 0.05 compared to EV control. ^#^*P* < 0.05 compared to palmitate control. ^&^*P* < 0.05 compared to insulin-untreated and palmitate-untreated group

Previous studies have shown that rosiglitazone, a potent PPARγ agonist, ameliorates insulin resistance in adipocytes^27^. Based on our findings demonstrating PPARγ activation by XBP1s, we investigated whether XBP1s could improve insulin resistance induced by treating adipocytes with palmitate (200 μM, 24 h), a well-known in vitro model for studying insulin resistance^28^. As shown in Fig. 6a, palmitate significantly inhibited insulin signaling by suppressing the levels of phosphorylated IRS1 and AKT. Insulin-mediated effects on GLUT4 cell surface translocation (Fig. 6b) and subsequent glucose uptake mostly disappeared in palmitate-treated adipocytes (Fig. 6c), indicating the establishment of a reliable in vitro insulin resistance model in adipocytes. However, upon palmitate treatment of XBP1s-overexpressing cells, the palmitate-induced suppression of insulin signaling and glucose uptake in adipocytes was significantly reversed (Fig. 6a–c).

PPARγ activation also exerts systemic insulin-sensitizing effects by regulating the expression of several genes involved in the synthesis of adipokines from adipose tissue^4^. As expected, intracellular mRNA and secreted protein levels of adipokines were altered in palmitate-treated adipocytes in our study. Namely, we observed a decrease in adiponectin and an increase in pro-inflammatory cytokines including leptin, MCP1 and TNFα (Fig. 6d, e). In accordance with these findings, expression levels of NFκB and P-JNK, two major signaling molecules that stimulate inflammation in adipocytes^29^, were also significantly increased upon palmitate treatment (Fig. 6f). In addition, glycerol release from adipocytes was significantly increased upon palmitate treatment, indicating that palmitate stimulates adipocyte lipolysis (Fig. 6g). However, just as we have observed above, ectopic XBP1s expression significantly reversed palmitate-induced dysregulation of adipokine expression and release (Fig. 6d, e), NFκB and P-JNK expression (Fig. 6f), and lipolysis (Fig. 6g). These findings strongly suggest that XBP1s has the potential to attenuate or reverse pathophysiological phenotypes associated with insulin resistance both in adipocytes and at the whole-body level.

## Discussion

In this study, we provide the first evidence that XBP1s is produced by insulin signaling and is an active mediator of insulin-stimulated glucose uptake in adipocytes through its effects on PPARγ activity. The effect of XBP1s on glucose uptake was completely abolished by a PPARγ inhibitor, suggesting that PPARγ activation is the exclusive mechanism by which XBP1s induces insulin sensitization in adipocytes. Of note, XBP1s regulation of PPARγ activity and the subsequent increase in glucose uptake by adipocytes were insulin-dependent; XBP1s-mediated increases in glucose uptake, GLUT4 translocation, PPARγ activity, and FGF21 expression were observed only in the presence of insulin. As GLUT4 translocation and glucose uptake in adipocytes are known to be regulated by PPARγ, these findings indicate that insulin signaling is necessary for XBP1s-induced PPARγ activation. Based both on our data and the present state of knowledge within the field, we propose a few possible mechanisms for this observation. First, increasing evidence demonstrates possible interactions between insulin signaling pathways and XBP1s. For example, XBP1s activates the phosphorylation of AKT, a key player in insulin-induced GLUT4 translocation in adipocytes^18,30^. Our results demonstrated that XBP1s increased the phosphorylation of both AKT and IRS1 in both insulin-sensitive and -resistant adipocytes. A recent report showing IRS1-mediated regulation of XBP1s protein stability in pancreatic β cells supports this assumption^31^. Second, insulin enhances XBP1s transcriptional activity by increasing its nuclear translocation in the livers of ob/ob mice^11^ and concordantly increases nuclear translocation and XBP1s target gene expression in adipocytes in our results. Third, insulin increases XBP1 mRNA splicing in adipocytes. Using two reporter assays that were specifically designed to directly measure XBP1 mRNA splicing and function, we clearly provided the first evidence that insulin enhances the generation of functionally active XBP1s through the phosphorylation of IRE1α. We also observed nearly complete abolition of the insulin-mediated effects on XBP1s, FGF21, PPARγ, and glucose uptake upon treatment with an IRE1α inhibitor, further supporting our conclusion. Considering that the levels of XBP1s are very low in the absence of insulin, these results imply that inducing the generation of XBP1s may be an indispensable additional step for insulin-mediated glucose uptake. Taken together, these findings strongly indicate that insulin sensitivity is maintained or amplified by a positive feedback mechanism involving XBP1s and insulin signaling molecules. Because XBP1s is ubiquitously expressed in our body, it would be interesting to investigate the role of XBP1s in insulin-sensitive tissues other than adipose tissue.

How does XBP1s increase PPARγ activity in response to insulin signaling? Currently, many molecules and mechanisms promoting PPARγ activity have been suggested^32^. Of these, we chose to investigate FGF21 because it was recently reported to be induced by XBP1s in the liver^22^. FGF21 is an inducible factor secreted mainly from the liver, adipose tissue, and skeletal muscle that exerts tissue-specific functions. In adipose tissue, FGF21 acts in a paracrine manner to promote insulin sensitivity by amplifying PPARγ activity^24^. A possible reciprocal mechanism by which PPARγ increases FGF21 expression in adipose tissue has also been reported^33^. However, the mechanism underlying this process remains unknown. Recently, it has been suggested that functional insulin signaling in adipose tissue is required for FGF21-mediated sensitization to insulin^25^, indicating the possibility that XBP1s may link insulin signaling and PPARγ activation by increasing FGF21 levels in adipocytes. In our study, we present the first evidence that XBP1s strongly amplifies the expression of FGF21, which plays a role in the insulin-induced activation of PPARγ in insulin-treated adipocytes. To the best of our knowledge, FGF21 does not affect the transcription of PPARγ; therefore, these findings indicate that the induction of FGF21 and the potential crosstalk between FGF21 and PPARγ is the main mechanism by which XBP1s mediates insulin-stimulated PPARγ activation and glucose uptake in adipocytes.

Saturated free fatty acids, such as palmitate, provoke inflammatory responses and cause insulin resistance in adipocytes through dysregulation of PPARγ^34^. In addition, PPARγ activation in adipose tissue and the resulting modulation of pro- and anti-inflammatory mediators has been widely accepted as an essential mechanism by which both glucose uptake and systemic insulin sensitivity can be improved^35,36^. Here, we have shown evidence that XBP1s alleviates palmitate-induced insulin resistance in adipocytes. XBP1s potently reversed palmitate-induced suppression of insulin signaling and dysregulation of adipokine expression, resulting in an increase in both the translocation of GLUT4 to the cell surface and glucose uptake in insulin-resistant adipocytes. In particular, XBP1s increased adiponectin, a well-recognized anti-diabetic adipokine, in both insulin-sensitive and -resistant cells. In this regard, Sha et al. recently reported that XBP1s promotes multimerization of adiponectin to form the most potent adiponectin isotype^15^. In addition, we also demonstrated that XBP1s strongly reduced fatty acid release from palmitate-treated adipocytes. To maintain systemic insulin sensitivity, it is important to reduce fatty acid release into the blood and the subsequent aberrant accumulation of lipids in non-adipose tissues. Considering that previous reports have demonstrated anti-lipolytic activities of FGF21 and PPARγ^37,38^, two downstream targets of XBP1s in our study, these findings suggest that the suppression of lipolysis is an additional potential mechanism of XBP1s-induced regulation of insulin resistance. The promotion of adipogenesis is an additional mechanism by which systemic insulin sensitivity is maintained; this process leads to a greater number of smaller adipocytes with increased lipid storage capacity and adiponectin production. Further, PPARγ has been widely accepted as a master regulator of adipogenesis. We have previously reported a few mechanisms by which XBP1s stimulates adipogenesis through increasing PPARγ expression in preadipocytes, implying that XBP1s may positively regulate systemic insulin sensitivity by increasing adipose tissue mass. In addition, PPARγ is suggested to regulate the inflammatory potential of adipose tissue macrophages, a central player in obesity-associated systemic insulin resistance^39^. Accordingly, PPARγ deletion or inactivation prevented anti-inflammatory polarization of macrophages and thus resulted in insulin resistance and glucose intolerance^39,40^. Interestingly, adipokines that were regulated by the XBP1s–PPARγ axis in our data, such as MCP1, TNFα, and leptin, are well-known factors regulating macrophage polarization^40,41^, indicating that XBP1s may regulate adipose tissue macrophages thorough a paracrine mechanism. Moreover, in our findings, XBP1s strongly suppressed the palmitate-induced phosphorylation of JNK and NFκB, two central signaling molecules driving M1 macrophage polarization^42,43^. Although we did not examine the role of XBP1s in macrophages in this study, it seems likely that XBP1s may play an anti-inflammatory role in this cell type. Taken together, these findings suggest that adipose XBP1s may control systemic glucose homeostasis and insulin sensitivity via a complex network of signaling pathways modulating adipokine secretion, lipolysis, adipogenesis, and the possible crosstalk between adipocytes and macrophages in adipose tissue.

**Fig. 7:**
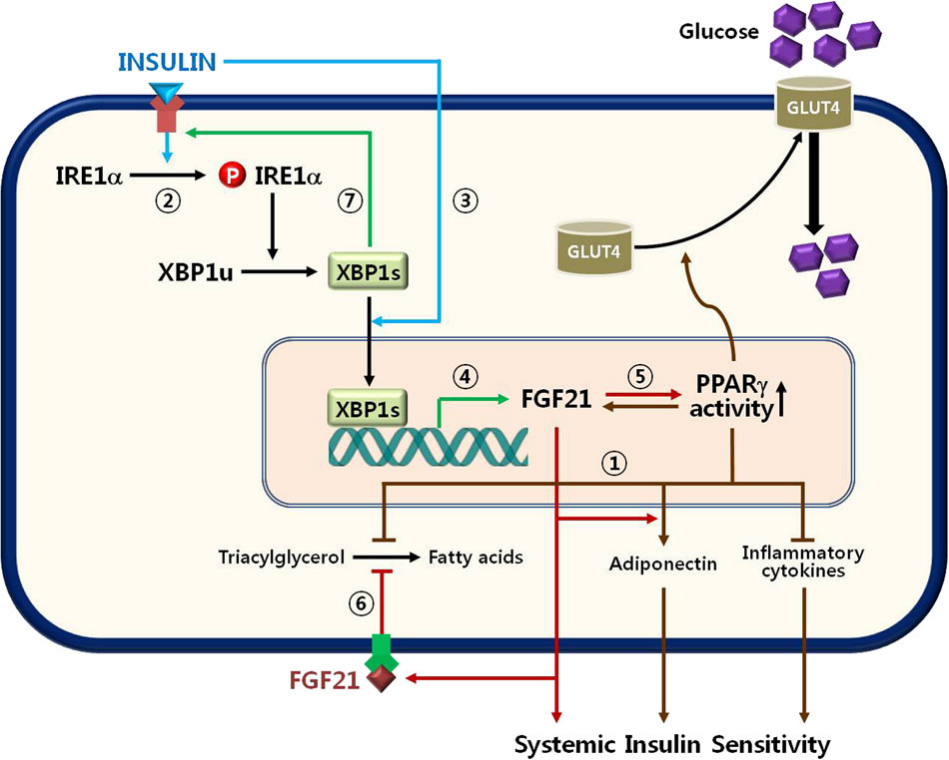
Schematic illustration of XBP1s-mediated regulation of insulin sensitivity in adipocytes. Accumulating evidence has indicated that PPARγ is a critical effector of insulin-sensitizing reactions such as GLUT4 translocation, adipokine secretion, and lipolysis (①). In this study, we suggest a novel mechanism underlying insulin-mediated PPARγ activation. XBP1s is generated and activated by the insulin-stimulated IRE1α–XBP1s signaling pathway (②) and the nuclear translocation of XBP1s (③). XBP1s induces the expression of FGF21 (④), which leads to PPARγ activation and vice versa (⑤), and directly inhibits lipolysis by an autocrine signaling mechanism (⑥). As a feed-forward amplifier, XBP1s also potentiates the insulin signaling cascade (⑦). Based on these effects, XBP1s strongly attenuates palmitate-induced insulin resistance in adipocytes. Refer to the Discussion section for a detailed explanation

In summary, XBP1s is generated and transcriptionally activated by insulin-mediated activation and nuclear translocation of IRE1α, thereby enhancing insulin-stimulated glucose uptake in mature adipocytes through a mechanism involving FGF21 induction and subsequent PPARγ activation (Fig. 7). XBP1s also regulates adipokine secretion and lipolysis in palmitate-treated adipocytes, suggesting a potential role for alleviating insulin resistance. When we combine these findings with the putative roles of XBP1s obtained from previous reports, it is very plausible that XBP1s is a critical factor for regulating insulin sensitivity in adipose tissue as well as at the whole-body level by enhancing PPARγ activity and/or expression.
